# Supplementary material for: Syndecan-1 Overexpressing Mesothelioma Cells Inhibit Proliferation, Wound Healing, and Tube Formation of Endothelial Cells
Source: Cancers (Basel). 2021 Feb 6;13(4):655. doi: 10.3390/cancers13040655 (PMC7915211; doi:10.3390/cancers13040655)
Supplement: Supplementary file 1 [file cancers-13-00655-s001.pdf]

cancers

MDPI

Supplemental Materials: Syndecan-1 Overexpressing Mesothelioma Cells Inhibit Proliferation, Wound Healing, and Tube Formation of Endothelial Cells

Joman Javadi, Ghazal Heidari-Hamedani, Angelika Schmalzl, Tünde Szatmári, Muzaffer Metintas, Pontus Aspenström, Anders Hjerpe and Katalin Dobra

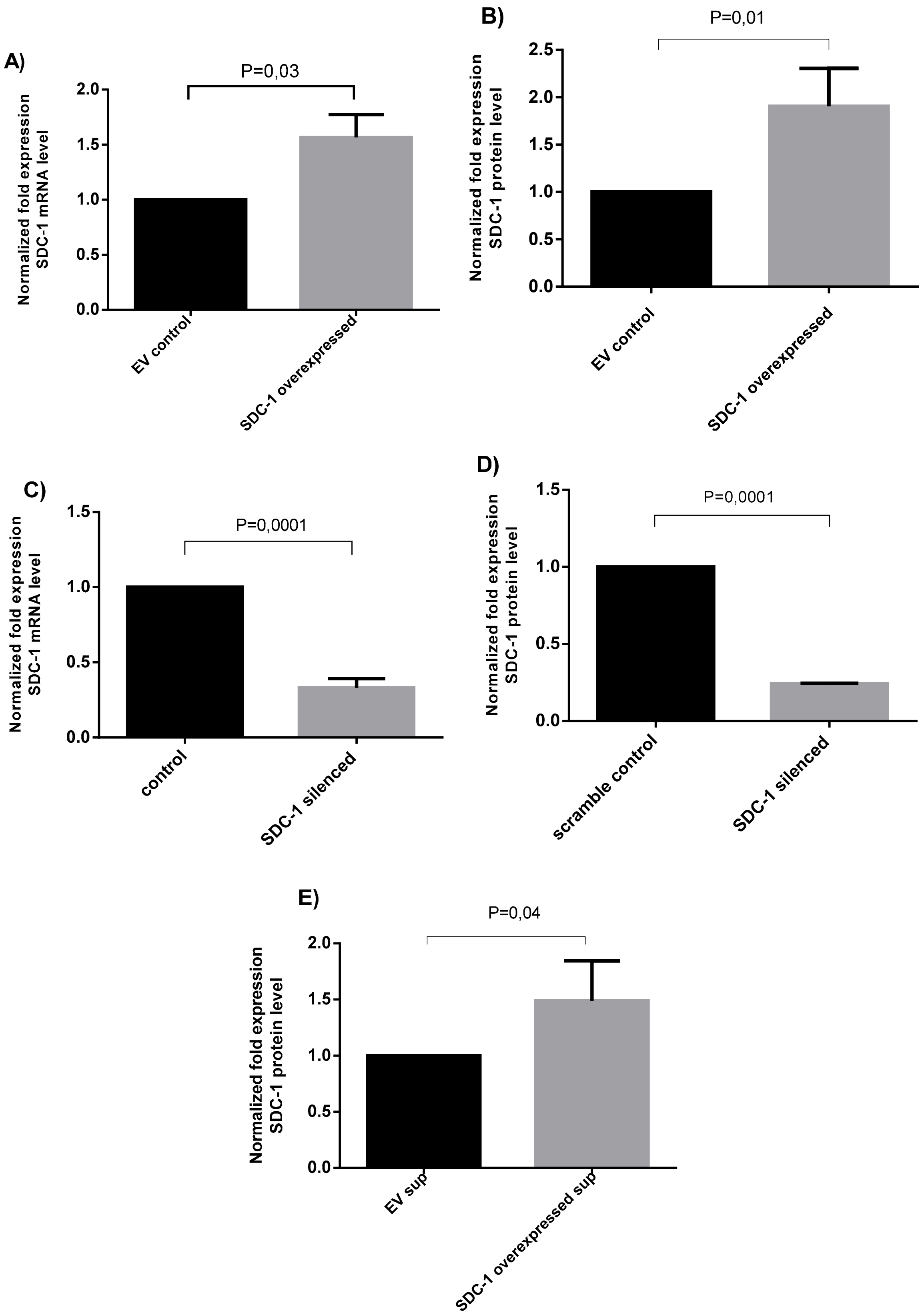

Supplementary Figure S1. Syndecan-1 (SDC-1) mRNA and protein expression level upon SDC-1 overexpression and silencing. SDC-1 mRNA level in SDC-1 overexpressed cells compared with empty vector (EV) control (**A**). The expression of SDC-1 was detected by qPCR and GAPDH was used as an internal control. SDC-1 protein level measured by ELISA in SDC-1 overexpressing cells compared with the EV control (**B**), SDC-1 mRNA level measured by qPCR in SDC-1 silenced

cells compared with the scrambled control (**C**), SDC-1 protein level measured by Luminex in SDC-1 silenced cells compared with scrambled control (**D**) and shed SDC-1 protein level in SDC-1 overexpressing cells supernatant (**E**). Columns represent Mean ± SEM of three independent experiments and data were analyzed with t-test to evaluate differences in SDC-1 expression ($p < 0.05$).

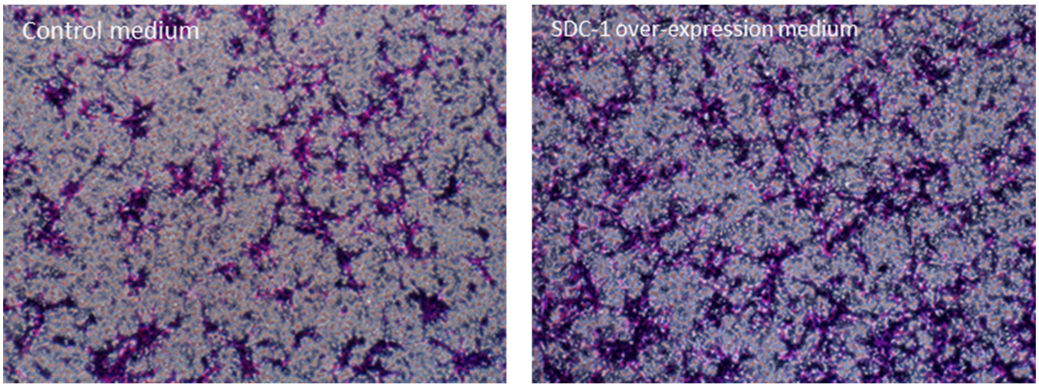

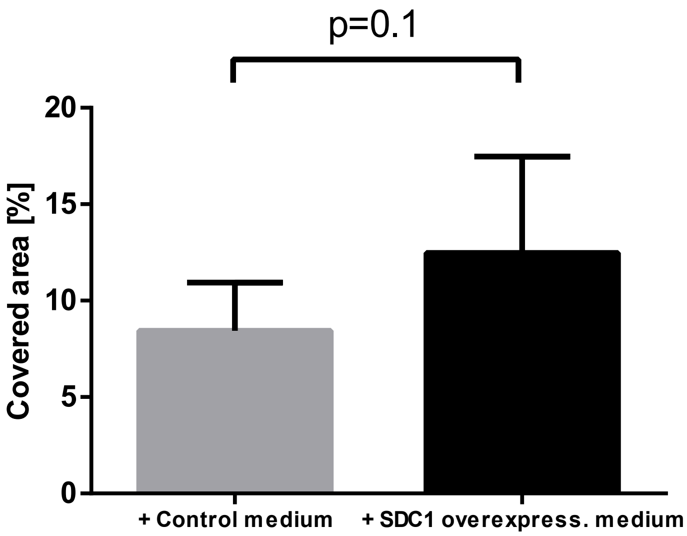

Supplementary Figure S2. Chemotactic directional movement of HUVEC cells toward the conditioned medium of malignant mesothelioma cells overexpressing SDC-1. Representative pictures of directional movement of endothelial cells. The percentage of total image area covered by HUVEC cells was determined by image analysis. Values show means of three independent experiments (± SD) conducted in duplicates. A paired t-test was performed to test the statistical significance at $p =< 0.05$.

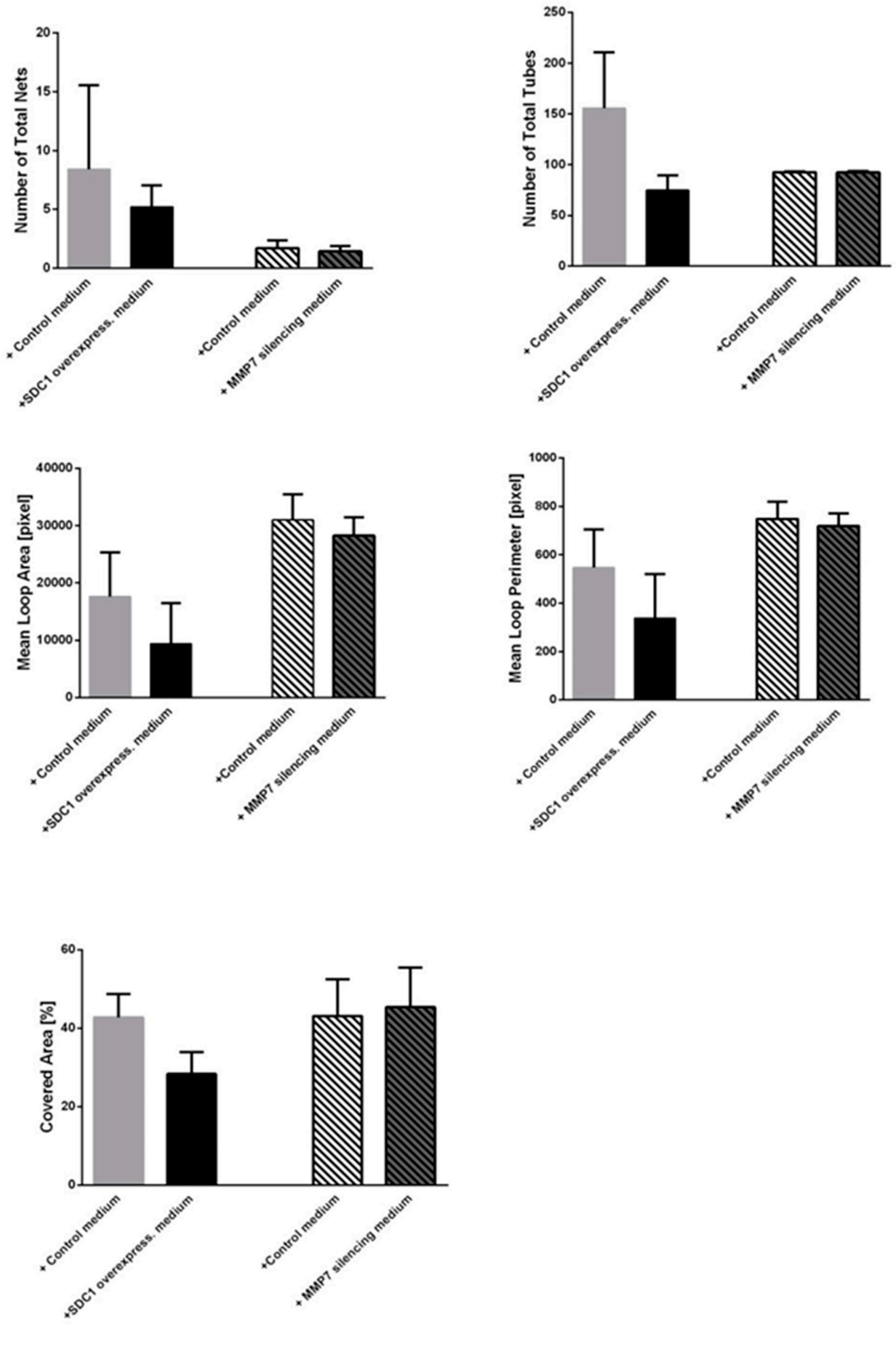

Supplementary Figure S3. Number of total nets and total tubes; mean loop area and perimeter, and percentage of covered area by tubes tended to be lower in HUVEC cells incubated with conditioned medium from SDC-1 over-expressing cells compared to corresponding controls. However, neither of these features did change when MMP7 was silenced. Two-tailed student's *t*-test was performed to test the statistical significance, where $p < 0.05$. Three independent experiments were performed. Values are mean ± SEM.

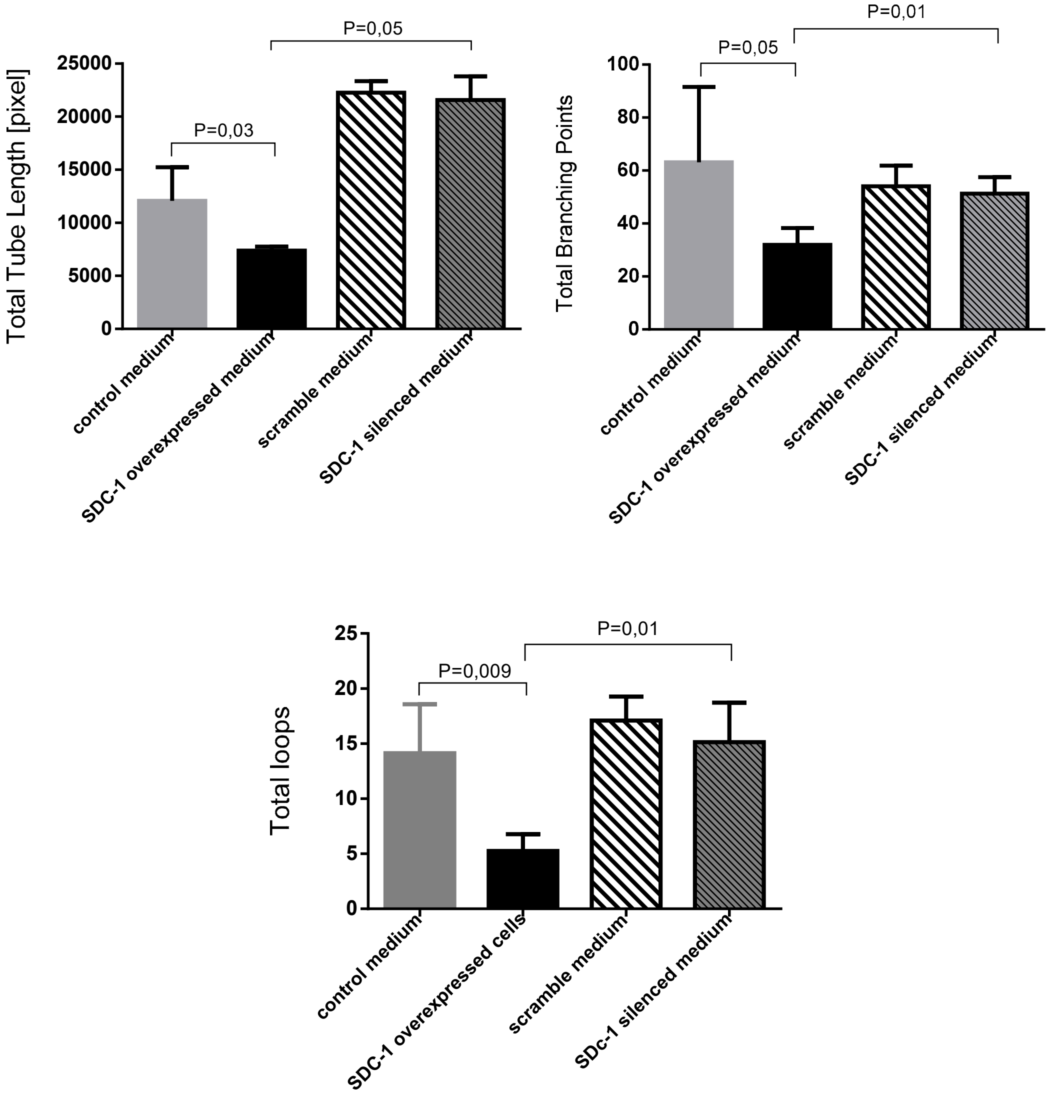

Supplementary Figure S4. Number of total tube length, total branching points and total loops are not significantly affected in HUVEC cells treated with conditioned medium from SDC-1 silenced cells compared to HUVEC cells treated with conditioned medium from negative silencer control (Sc). Conditioned medium from SDC-1 silenced malignant mesothelioma cells does not inhibit tube formation of HUVEC cells. A) Total tube length B) Total branching points C) Total loops. Two-tailed student's *t*-test was performed to test the statistical significance, where $p < 0.05$. Three independent experiments were performed. Values are mean ± SEM.

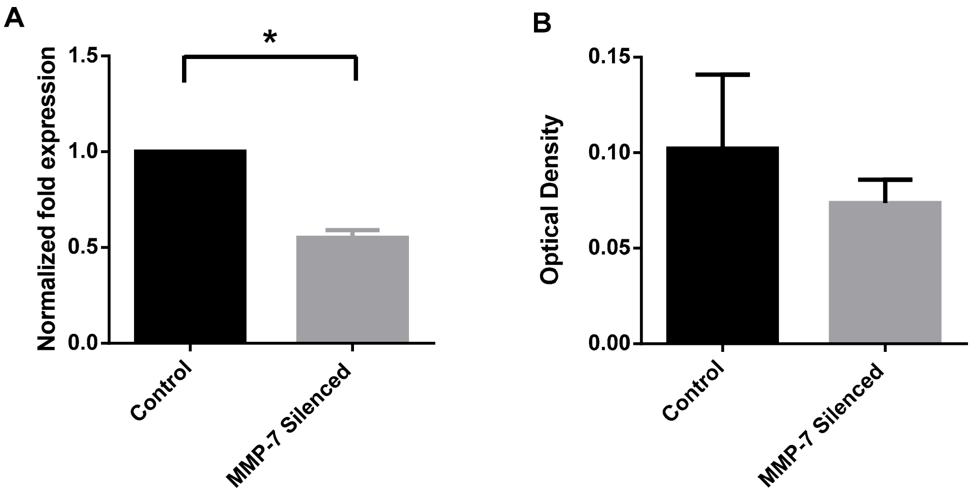

Supplementary Figure S5. MMP-7 expression level of malignant mesothelioma cells after MMP7 silencing and its effect of SDC-1 shedding. mRNA levels of MMP-7 after silencing compared to scrambled control (A). Shed SDC-1 levels in supernatant from MMP-7 silenced malignant mesothelioma cells compared to scrambled control (B). Shed SDC-1 level was measured by ELISA. Values show means of three independent experiments (± SD). Two-tailed student's *t*-test was performed to test the statistical significance, where $p < 0.05$. Three independent experiments were performed. Values are mean ± SEM.
